# Association of the Human Bocavirus With Tonsil Squamous Cell Carcinomas

**DOI:** 10.3389/fmicb.2018.02450

**Published:** 2018-10-16

**Authors:** Merle Höpken, Isabel Förster, Steffen Maune, Michael Brockmann, Oliver Schildgen, Verena Schildgen

**Affiliations:** ^1^Klinik für Hals-, Nasen- und Ohrenheilkunde, Kliniken der Stadt Köln, Klinikum der Privaten Universität Witten/Herdecke mit Sitz in Köln, Cologne, Germany; ^2^Institut für Pathologie, Kliniken der Stadt Köln, Klinikum der Privaten Universität Witten/Herdecke mit Sitz in Köln, Cologne, Germany

**Keywords:** human bocavirus, tonsil squamous cell carcinoma, oncogenesis, persistence, primary cell culture

## Abstract

**Background:** The human bocavirus (HBoV) is known to persist latently in the infected host cells and seems to replicate its DNA via the DNA damage response system, which is frequently defect in tumors and correlates with microsatellite instability (MSI). Because HBoV is able to persist in the infected tissues, induces pro-fibrotic and pro- cancerogenic cytokines *in vivo* and *in vitro*, and is detected in colorectal and lung tumors, the virus may be involved in cancerogenesis at least as a cofactor. Recently it was shown that the adenotonsillar tissue is an important site of HBoV1 persistence and replication. Considering the background that approximately 60% of oropharyngeal cancers were thought to be attributable to a HPV infection, a co-participation of HBoV in terms of a chronic virus infection might play a role in the cancerogenesis of tonsil tumors.

**Methods:** Formalin-fixed, paraffin-embedded tonsil tumor samples were screened for HBoV and HPV DNA. Positive tissue sections were afterward subjected to fluorescence *in situ* hybridization (FISH) analysis to identify HBoV and HPV infected cells. By use of an *in vitro* cell culture model with primary tonsil fibroblasts, keratinocytes, and lymphocytes infected by HBoV we tried to find the target cells of virus replication. MSI testing was based on a previously published protocol using a de-multiplexed PCR followed by fluorescent detection of PCR products in a capillary sequencing device.

**Results:** In total 62 of 103 (60, 19%) of the tonsil squamous cell carcinomas tested positive for HBoV DNA and 66 of 103 (66%) samples were identified as HPV positive. The FISH analysis revealed both double infection of HPV and HBoV in the same cells as well as single infections of both viruses within the tumor tissue. Twenty-two of 62 HBoV positive tumors tested HPV negative, 40 of 62 tissue sections were HBoV and HPV positive. We analyzed 21 out of the 62 HBoV positive tumors for MSI. Of those four tonsils displayed MSI in at least 1 of 10 microsatellite markers.

**Conclusion:** Our findings support the hypothesis that human bocavirus infections as a cofactor may have an impact on tumor development in tonsils, although it still remains possible that HBoV solely displays a tumor tropism.

## Introduction

Since the identification of human bocavirus 1 in nasopharyngeal aspirates in 2005, evidence is increasing that the HBoV1, belonging to the *Bocaparvovirus* genus of the *Parvoviridae* family, is associated with respiratory tract infections and gastrointestinal infections (Allander et al., 2005; Huang et al., 2012). It is presumed that besides causing acute infections HBoV is persisting in a replicative but subclinical state, and is a pathogen rather than an innocent bystander (Proenca-Modena et al., 2014). Previous studies suggest that HBoV is associated with some lung and colorectal tumors and prove that the bocavirus persistence is increased in cancer patients (Schildgen et al., 2013). In 20% of colorectal and lung cancers formalin-fixed paraffin-embedded (FFPE) tissues human bocavirus DNA could be detected. Li et al. (2007) revealed, that HBoV DNA commonly occurs in the serum of cancer patients and that the prevalence is significantly higher in this group than in that of the healthy control group (Addie et al., 2012). It is also known that HBoV persists in adenotonsillar tissue, which is considered as the major replication site *in vivo* by some researchers (Clement et al., 2009). Proenca-Modena et al. (2014) detected human bocavirus in 31.1% of 172 hypertrophic adenoids or tonsils from children. Szalmas et al. (2013) reported that the presence of HBoV displays a statistically significant correlation to Otitis media with effusion cases associated with adenoid hypertrophy. It could be shown that HBoV1 can persist in the infected host in form of cccDNA structures, similar to the human hepatitis B virus (HBV), leading to a chronic inflammation and culminating in organ-fibrosis (Lusebrink et al., 2011; Schildgen et al., 2014).

This observations are supported by a preceding study, which demonstrated that profibrotic and pro-cancerogenic chemokines are upregulated *in vitro* and *in vivo*, suggesting that HBoV is directly or indirectly involved in tumorigenesis (Khalfaoui et al., 2016). In addition, given the fact that the incidence of human papillomavirus associated oropharyngeal cancer is increasing especially in developed countries, the search for infectious agents playing a role in human carcinogenesis in the range of the oropharyngeal tract and their identification are important issues (Marur and Forastiere, 2008). In the case of HPV infection of the oropharynx it is presumed that the process toward malignant conversion is covering a long latency period between primary infection and cancer emergence (zur Hausen, 1999). Due to the latent, chronic inflammation provoked by oncogenic viruses this mechanism could be similar in the presence of a chronic HBoV infection (Wang et al., 2008; Zeisel et al., 2015). As animal models are still lacking for the exploration of HBoV infections, cell culture experiments and the analysis of clinical cohorts are necessary to elucidate the pathophysiology of replication, interaction with cells and other viruses and possible oncogenic effects. Taken together the previous findings lead us to the hypothesis that HBoV also persists in adult tonsillar tissue and may be even associated with oropharyngeal, especially tonsillar cancers.

## Materials and Methods

Aim of the present study was to address the questions whether HBoV can be detected in tonsillar squamous cell carcinomas, whether there is a correlation of HPV and HBoV infection in tonsillar tumors and whether both viruses infect the same cells. Furthermore, we cultivated primary keratinocytes, fibroblasts, and lymphocytes of tonsil tissue and infected them with virulent HBoV particles to ascertain which the target cells for virus replication are. In addition, we tested the hypothesis that the interaction between HBoV and the host genome, in concert with the fact that the viral replication is dependent on the DNA damage repair response (Deng et al., 2016, 2017), may influence the microsatellite stability in tonsillar tumors.

### Ethics Statement

All procedures were performed in accordance with the declaration of Helsinki and according to a vote of the ethical committee of the Private University of Witten/Herdecke (Vote No. 151/2016). This vote was specifically approved for the current study. There is a written informed consent of all patients, whose primary cells were used. Pediatric patients were included exclusively with parental written informed consent. For the retrospective and double blinded part of the study no written consent was required. The study cohort consisted of adult patients (tumors and primary cells) and children (primary cells).

### Patient Samples

Hundred and three FFPE tonsillar cancer sections and 20 chronically inflamed tonsil sections were selected from archived samples from our routine clinical laboratory. Primary tonsil cells were gained by routinely performed tonsillectomy in adult patients (*n* = 11).

### PCR Based Detection of Viral Nucleic Acids

DNA from FFPE tissue samples of 103 tonsillar cancers and 20 chronic inflamed tonsils was extracted using the Maxwell 16 FFPE tissue kit (Promega, Mannheim, Germany) according to the manufacturer’s protocol. All samples were analyzed with the RespiFinder Smart22 (Pathofinder, Netherlands) being able to detect 22 respiratory pathogens, including HBoV and Adenovirus. HPV detection was performed by the HPV 3.5 LCD-Array (Chipron, Berlin, Germany) according to the manufacturer recommendations. HBoV real-time PCR was performed as previously described (Khalfaoui et al., 2016).

### HBoV and HPV Detection by FISH Analysis

All FFPE tonsil tumor samples that tested positive for HBoV by the Respifinder assay (*n* = 62) were subjected to FISH analyses for the detection of HBoV1 and HPV 16.

Fluorescence *in situ* hybridization was performed as follows: FFPE tumor tissues were cut into slices of 3-μm and mounted on slides. Slides were treated according to the ZytoLight SPEC ALK/EML4 TriCHeck probe protocol (ZytoVision, Bremerhaven, Germany) following the manufacturers’ recommendations as previously published (Schildgen et al., 2013), with the modification that HBoV and HPV specific probes were mixed in a ratio of 1:1 (2 pM each). The HBoV probe was designed to hybridize near the terminal region of the HBoV genome and labeled with rhodamine red (Eurofins MWG Operon, Ebersberg, Germany). To identify the genome of the human papilloma virus 16 a commercially available biotin labeled HPV 16 probe (PanPath, Budel, Netherlands) was used. At this point the protocol was modified again, because the tissue was then incubated in Avidin-FITC (PanPath, Budel, Netherlands) for 30 min at 37°C before counterstaining of the cell nuclei with DAPI (DCS, Hamburg, Germany) to make HPV fluorescently visible.

Microscopic analyses and documentation was performed on a Zeiss Axioplan microscope using the AxioVixion 4.8 software (Zeiss, Jena, Germany).

### Cell Culture

All cultures were performed with 1% v/v Penicillin/Streptomycin (10^4^ U/ml each, BioWhittaker/Lonza, Switzerland) at 37°C. Human embryonic kidney 293 (HEK 293) cells for virus production were obtained from American Type Culture Collection (ATCC via LGC Standards, Wesel, Germany) and were cultured according to ATCC’s recommendations. Primary tonsil keratinocytes, fibroblasts, and lymphocytes were obtained from 11 patients suffering from chronic tonsillitis immediately after routine tonsillectomy. Written informed consent was obtained in all cases in agreement with the ethical vote for this study. Resected tonsils were directly placed in 37°C warm keratinocyte growth medium (KGM BulletKit, Lonza, Switzerland). Further on lymphatic follicles and epithelial tissues were dissected using a scalpel. Afterward, the epithelial sections of the tonsils were minced in a Petri dish and were then enzymatically dissociated in trypsin for 15 min before being passed through an EASYstrainer cell filter (Greiner, Frickenhausen, Germany). The lymphocyte section was treated with trypsin for 5 min and was also passed through an EASYstrainer cell filter. While lymphocytes were cultured in six-well plates as suspension cultures in RPMI medium, keratinocytes and fibroblasts were cultured on collagen-coated six-well plates (coated with 1 ml of 30 mg collagen solved in 500 ml deionized water and 100 μl glacial acetic acid) grown in keratinocyte growth medium (KGM BulletKit, Lonza, Switzerland) or epithelial growth medium (EGM BulletKit, Lonza, Switzerland), respectively. Microscopic analyses and documentation were performed on a Zeiss Primo Vert microscope using the AxioVision 4.8 software (Zeiss, Jena, Germany).

### Virus Infection

Infectious human bocavirus was produced and quantified essentially as described earlier by Huang et al. (2012). The infection of the tonsillar cultures was performed in 90% confluent fibroblast and keratinocytes cell dishes, which was reached after 2–3 weeks of cultivation. The fibroblast infection was performed in triplicate, whereas the keratinocyte infection was conducted twice (in triplicate for one patient and as single infection for a second patient). The fibroblasts and keratinocytes were infected at MOI 0.5 with HBoV1 Geq in 3 ml cell medium, released from the apical surface of HEK293 cells after transfection of the full length HBoV1 plasmid pIHBoV1. The lymphocyte suspension cultures were infected at MOI 0.5 with HBoV1 inoculum immediately after preparation. Lymphocytes were previously counted by Luna-FL Fluorescence cell counter (Logos Biosystems, South Korea). The lymphocyte infection was also performed in triplicate (9 lymphocyte cultures, obtained from three different patients). Fibroblasts were harvested 12 days, keratinocytes and lymphocytes 10 days post-infection. The supernatant of the keratinocyte culture was removed and kept for quantitative HBoV1 PCR on days 1, 2, 3, 4, 5, 6, 7, and 8 following infection. The supernatant of the fibroblast culture was harvested on days 1, 3, 6, and 12 following infection, the lymphocyte culture supernatant on days 1, 3, 6, 8, and 10 following infection. HBoV DNA was extracted with the Maxwell RNA kit, which allows simultaneous DNA and RNA extractions from liquid samples (Promega, Darmstadt, Germany). Afterward the supernatant and the harvested cells were subjected to a real-time HBoV-1 PCR for a quantitative analysis of viral genomes.

### Microsatellite Instability

Microsatellite instability testing was based on a previously published protocol using a multiplex PCR followed by fluorescent detection of PCR products in a capillary sequencing device. Ten microsatellites were included in the protocol, namely BAT25, BAT26, BAT40, APC, D17s250, D2s123, D13s153, D18s58, D10s197, and MycI. The protocol was de-multiplexed and established on the QiaXcel Advanced system (Qiagen, Hilden, Germany) (Forster et al., 2018).

### Statistical Analyses

In order to test if a higher tumor stage correlates with the detection of HBoV, Pearson correlation coefficient r_Pearson_ and the coefficient of determination r^2^, and the statistical significance were calculated with the one-sided Student’s *t*-test and a chi-square test.

## Results

**FIGURE 1:**
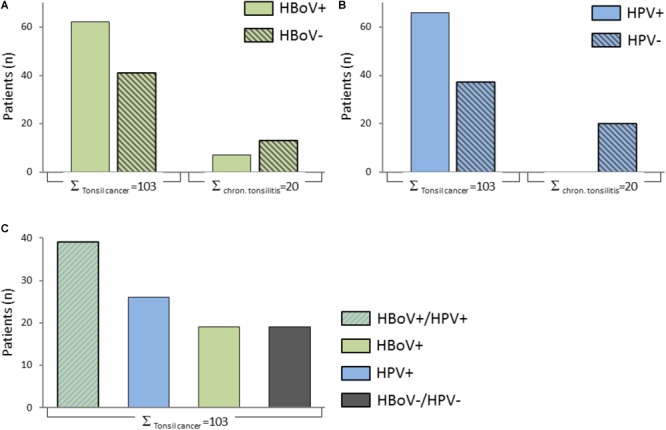
Column diagrams showing the overview on the patient cohort. **(A)** Shows the rate of HBoV positivity in the tumor cohort compared to patients with chronic tonsilitis. **(B)** Shows the rate of HPV positivity in the tumor cohort compared to patients with chronic tonsillitis. **(C)** Shows the rate of HBoV/HPV coinfections and mono-infections in the entire tumor cohort.

In total, 62 of 103 (60.19%) of tonsil squamous cell carcinomas and 7 of 20 (35%) of chronically inflamed tonsils tested positive for HBoV DNA by the RespiFinder Smart22 PCR (**Figure 1A**). In 66 of 103 (66%) tonsil tumors, but in none (0%) of the 20 chronically inflamed tonsils HPV DNA could be identified (**Figure 1B**). In 40 of 103 (38.8%) tonsil tumors there was a coinfection of HPV and HBoV. In 26 of 103 (25.2%) we could register a single HPV infection and in 19 of 103 (18.4%) a single HBoV infection (**Figure 1C**). All samples were negative for the other 21 airway pathogens tested by the RespiFinder assay.

**FIGURE 2:**
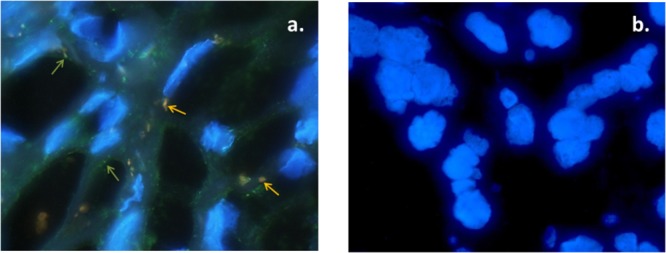
Examples of FISH analysis of a tonsillar tumor being positive for HPV and HBoV by RespifinderSmart22 and the HPV 3.5 LCD-Array. Rhodamine red indicates the human bocavirus DNA, whereas FITC indicates human papillomavirus 16 DNA. The blue DAPI staining indicates the cell nuclei. **(a)** HBoV positive tumor. **(b)** HBoV negative tumor.

To ascertain whether HPV and HBoV DNA are present in the same cells of the tumor tissue an HBoV specific FISH assay was applied (**Figure 2**). The HPV type 16 FISH assay established for routine diagnostic was in agreement with the results of the HPV PCR, too. In 24 of 40 co-infected tumor sections (60%) the fluorescent signals of HPV 16 and HBoV DNA could be detected in the same cells, implying that the two viruses infected the same host cell. In contrast we found virus persistence in different cells in 13 of 40 co-infected tumor samples (32.5%) which means that FITC signals (indicating HPV DNA) and rhodamine red signals (indicating HBoV 1 DNA) were spread as distinct separated signals all over the tumor sections.

In 3 of 40 tumors (7.5%) we could observe a heterogeneous image with double infected cells and single infections in the same section. In 22 cases (35.4%) of all HBoV positive samples (single and co-infections) the presence of human bocavirus DNA could be determined. In accordance with the PCR based analyses HPV DNA was not detected in the chronic tonsillitis tissue sections. The signals were not always located in the nucleus, but appeared to be in the cytoplasm, as indicated by merging the different color channels. Moreover, multiple signals per cell were registered, indicating that multiple copies of HBoV DNA are present in the cells due to replication or, as a matter of speculation, due to persisting episomes.

Furthermore we found a correlation between the presence of human bocavirus and the tonsil tumor stage. We noticed higher bocavirus prevalence in tumor sections with progressed tumor stages. By using the one-tailed *t*-test for two dependent means a trend for the increase in the number of HBoV-positives in higher tumor stages was observed (value of *p* = 0.09051 at *p* ≤ 0.10 in the HBoV positive group vs. *p* = 0.114027 at *p* ≤ 0.10 in the HBoV negative group) (**Table 1A**), whereas there was no higher HPV prevalence in higher tumor stages (**Table 1B**).

**Table 1:** Case numbers and percentages of HBoV **(A)** and HPV **(B)** according to tumor stages.

(A)	HBoV +	HBoV -
Stage 1	5 (41.7%)	7 (58.3%)
Stage 2	2 (28.5%)	5 (71.5%)
Stage 3	17 (68.0%)	8 (32.0%)
Stage 4a	30 (54.5%)	25 (45.5%)
Stage 4b	3 (100%)	0 (0%)
*Pearson correlation coefficient r*_*Pearson*_ = *0.913; coefficient of determinationr*^*2*^ = *0.8335; very high correlation*.		
**(B)**	**HPV +**	**HPV -**

Stage 1	4 (33.3%)	8 (66.7%)
Stage 2	5 (83.3%)	1 (16.7%)
Stage 3	16 (66.7%)	8 (33.3%)
Stage 4a	37 (64.9%)	20 (35.1%)
Stage 4b	3 (100%)	0 (100%)

*Pearson correlation coefficient r*_*Pearson*_ = 0.9213; *coefficient of determination r^*2*^ = 0.8487; very high correlation*.

*By using the one-tailed *t*-test for two dependent means there is a trend for an increase in the number of HBoV-positives in higher tumor stages (value of *p* = 0.09051 at *p* ≤ 0.10 in the HBoV positive group vs. *p* = 0.114027 at *p* ≤ 0.10 in the HBoV negative group).*

**FIGURE 3:**
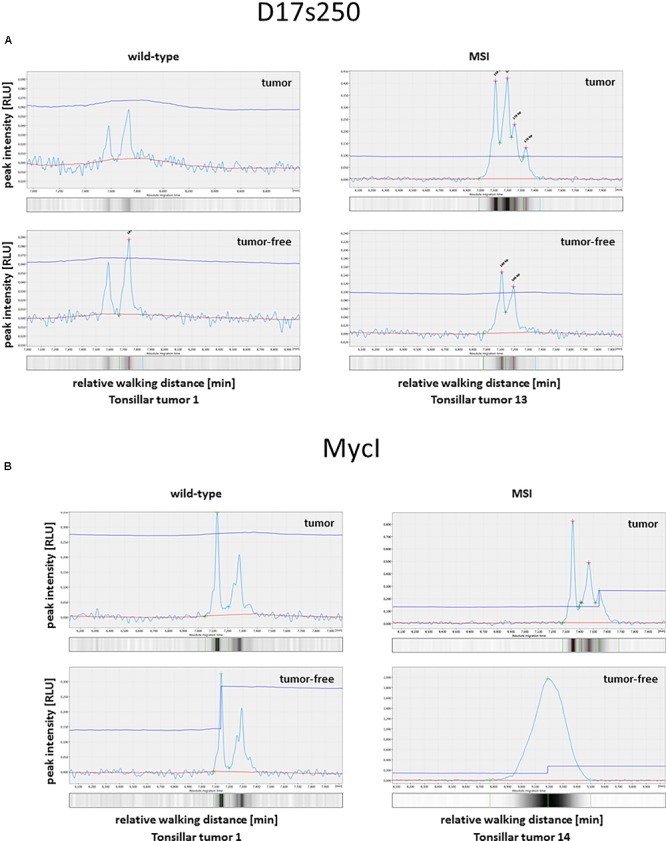
Analyses of microsatellite instability in tonsillar tumors. The electropherograms show selected microsatellites in tumor and the corresponding healthy control tissue. **(A)** MSI for D17s250. **(B)** MSI for MycI.

In addition, we analyzed 21 out of the 62 HBoV positive tumors (single HBoV infected and HPV coinfected) for MSI (**Table 2**), because only for these tumors a tumor-free control tissue was available to compare the microsatellite status of tumor and otherwise healthy tissue. Of those, only four tonsils displayed microsatellite instabilities in at least 1 of 10 microsatellite markers, as exemplarily shown in **Figure 3**.

**Table 2:** Overview on the results of MSI testing in 21 HBoV positive tumor samples eligible for the analyses.

HBoV (+ tonsillar tumor	BAT 25	BAT 26	APC	D17s250	D2s123	D13s153	BAT 40	Mycl	D18s58	D10s197
1	wt	wt	wt	wt	wt	wt	wt	wt	wt	wt
2	wt	wt	wt	wt	wt	wt	wt	wt	wt	wt
3	wt	wt	wt	wt	wt	wt	wt	wt	wt	wt
4	wt	wt	wt	wt	wt	wt	wt	wt	wt	wt
5	wt	instable	wt	wt	instable	instable	wt	wt	instable	wt
6	wt	wt	wt	wt	wt	wt	wt	wt	wt	wt
7	wt	wt	wt	wt	wt	wt	wt	wt	wt	wt
8	wt	wt	wt	wt	wt	wt	wt	wt	wt	wt
9	wt	wt	wt	wt	wt	wt	wt	wt	wt	wt
10	wt	wt	wt	wt	wt	wt	wt	wt	wt	wt
11	wt	wt	wt	wt	wt	wt	wt	wt	wt	wt
12	wt	wt	wt	wt	wt	wt	wt	wt	wt	wt
13	wt	wt	wt	instable	instable	wt	wt	wt	wt	wt
14	wt	wt	wt	wt	wt	wt	wt	instable	wt	wt
15	wt	wt	wt	wt	wt	wt	wt	wt	wt	wt
16	wt	wt	wt	wt	wt	wt	wt	wt	wt	wt
17	wt	wt	wt	wt	wt	wt	wt	wt	wt	wt
18	wt	wt	wt	wt	wt	wt	wt	wt	wt	wt
19	wt	wt	wt	wt	wt	wt	wt	wt	wt	wt
20	wt	wt	wt	wt	wt	instable	wt	wt	wt	wt
21	wt	wt	wt	wt	wt	wt	wt	wt	wt	wt

*wt, wild type.*

**FIGURE 4:**
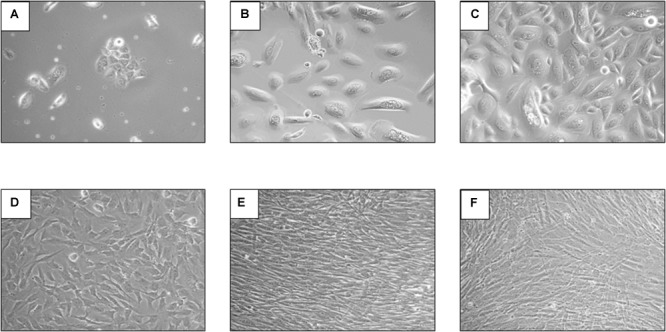
Microscopic pictures were taken with 100x magnification with phase contrast. The upper part of the figure shows keratinocytes of the tonsil 8 days **(A)**, 3 weeks **(B)**, and 4 weeks **(C)** after cultivation in KGM medium. The lower pictures show fibroblasts of the tonsil 5 days **(D)**, 10 days **(E)**, and 14 days **(F)** after cultivation in KGM medium.

**FIGURE 5:**
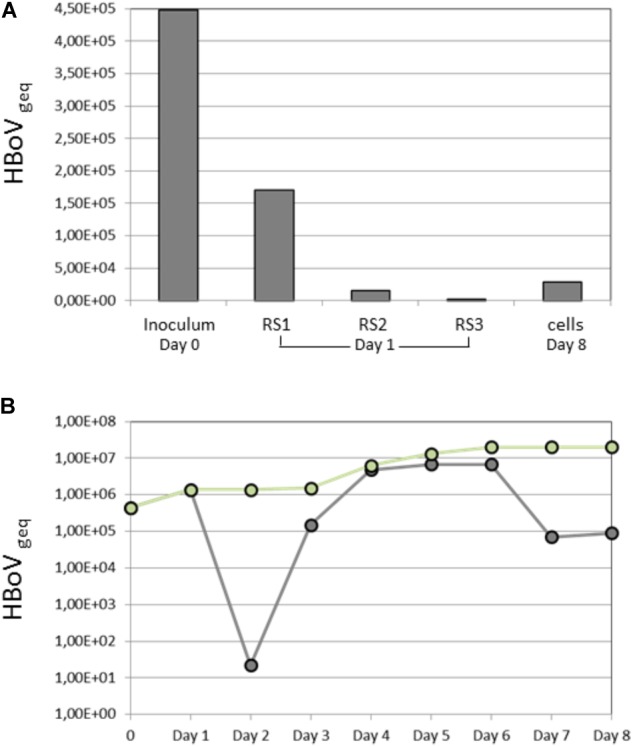
Virus kinetics following infection of keratinocytes with HBoV. The supernatant was harvested from day 1 to day 8 post-infection. **(A)** Shows the amount of HBoV (geq) in the inoculum as well as in the three rinsing steps (RS1-3) at day 1 after infection. Finally, 2.8 × 10^4^ HBoV geq were detected cell-bound at day 8. **(B)** Shows the accumulated viral load (geq) (green) and the daily produced viral load (geq) (gray) in the supernatant, respectively.

Because it was postulated that HBoV is likely persisting in tonsillar tissue and that this kind of tissue is assumed to be the major replication site *in vivo*, we performed a series of cell culture experiments with primary tonsil cells, directly obtained after tonsillectomy. Routinely resected, chronic infected tonsils were brought to the laboratory immediately after surgery and fibroblasts, keratinocytes, and lymphocytes were prepared. To evaluate the most appropriate coating keratinocytes were cultured on laminin, fibronectin, and collagen coated plates and it turned out that collagen is the most effective coating for tonsillar keratinocytes. Nevertheless cultivating of tonsil keratinocytes was successful in solely 2 of 11 patients. These cells were infected with an HBoV inoculum with MOI 1 referred to Geq in triplicate and the amount of virus genome was determined kinetically up to day 12. Regarding the cell morphology of the isolated keratinocytes and fibroblasts, which is exemplarily shown in **Figure 4**, no difference between infected and non-infected cells could be observed (not shown). In one infected well of one patient we observed a virus replication after 3 days in accordance with the typical virus growth curve (**Figure 5**). However, in the remaining three HBoV infected cultures of the other patient there was no virus replication measurable. Virus replication in fibroblasts and lymphocytes was not detectable, indicating that this cell types are not permissive for HBoV.

## Discussion

It was postulated that HBoV persists in tonsillar tissues which are deemed to be the major replication site during the natural infection *in vivo* (Clement et al., 2009). Therefore, we addressed the question of HBoV also plays a role in tonsillar carcinoma.

In our study including 103 tonsil squamous cell carcinomas and 20 chronic infected tonsils, we detected human bocavirus DNA in 60.2% (*n* = 62) of all tonsillar tumors and in 37% (*n* = 7) of all chronic tonsillitis tissues. The data presented suggest that there is an association between the presence of HBoV and oropharyngeal squamous cell cancer, especially tonsillar carcinomas. The prevalence of HBoV is statistically significant higher in tonsillar tumor sections than in chronically inflamed tonsils (*p* = 0.037 by Chi-square test), which not only confirms the hypothesis that HBoV may be a causative pathogen in coinfection, but as well might imply that HBoV may be involved in cancerogenesis. Up to now it is not clearly verified whether HBoV persists in tumors as episomes in form of cccDNA, or whether it is integrated into the human genome but there are several studies concluding that the cccDNA structures, found in patients infected with HBoV may be viral genomes persisting in the cells (Kapoor et al., 2011; Lusebrink et al., 2011). This mechanism would be similar to the human hepatitis B virus (HBV), a virus with high oncogenic potential because of its capacity to induce chronic inflammation, leading to liver fibrosis and consequently supporting cancerogenesis (Schildgen et al., 2014). We could register multiple copies of HBoV DNA in the tumor cells due to replication or, as a matter of speculation, due to persisting episomes. This observation fits with our earlier observations when analyzing non-small lung cancers and colorectal tumors (Schildgen et al., 2013). Additionally, the role of human parvovirus as a potential contributor to carcinogenesis has been discussed for parvovirus B19 with the reasonable suspicion that the virus is a causative agent of lung and colon adenocarcinomas due to chronic inflammation (Tabata et al., 2005; Li et al., 2007). We could carefully speculate that HBoV as a single stranded DNA virus, persisting probably in form of cccDNA structures, can act in a similar way or utilizes related mechanisms like HBV. As no further respiratory pathogens were detected in the tonsillar tumors, the likelihood of a causal involvement of HBoV in the development of tonsillar tumors is increased.

It is known that the incidence of human papillomavirus-associated oropharyngeal cancer is increasing especially in developed countries up to 60 to 80% (Kim, 2016; Marur and Forastiere, 2016). This is why we analyzed and compared the presence of HPV in conjunction with HBoV DNA in tonsillar tumor tissue. PCR analyses of our study revealed a coinfection rate of 37.8% in tonsillar carcinomas and our data show that in 60% of these HPV and HBoV positive tumors the viral genomes occur within the same cells. Taking into account that it is not known which infection take place initially, this may mean that synergistic effects arise, which change the mode of cancerogenesis.

Further, we hypothesized that due to the fact that HBoV infections make use and modulate the DNA damage repair systems, HBoV positive tumors could be associated with increased MSI. Thereby, we assumed that especially if the viral DNA would integrate into the host’s genome MSI could be more frequently. Our data, in contrast, demonstrate that HBoV-positivity of tonsillar tumors does not go ahead with MSI, thus there appears to be no such interaction.

Regarding the target cells for infection it appears that lymphocytes and fibroblasts do not seem to be permissive for the virus, as we confirmed in triplicate in cultured fibroblasts and lymphocytes. As we could observe virus replication following HBoV infection of cultured keratinocytes in only one patient, this data must be interpreted very carefully due to the insufficient number of experiment repetitions, but they may give a first hint that keratinocytes represent a HBoV permissive cell in the tonsil. The lack of repetitions of the infection was mainly caused by the fact that culturable keratinocytes could only be isolated to a sufficient extent from 2 of 11 tonsils, of which only cells from one patient were permissive for the virus.

The observation that HBoV can persist in infected tonsil squamous cell carcinomas and chronic infected tonsils, similar to other oncogenic viruses, has to be discussed from different perspectives. One could indeed speculate that HBoV is only a harmless bystander, which just benefits of accelerated cell proliferation. The hypothesis that HBoV solely uses the cancer cells as their place of replication is supported by previous observations made with other parvoviruses. The majority of known parvoviruses is dependent on the cell cycle and are capable of replicating, probably exclusively, in actively replicating cells (in S phase). For this reason, they thrive in cancerous cells, often giving the false impression of being oncogenic (Canuti et al., 2014). In addition, there is indeed a well-established relationship between cancer and parvoviruses but this is most likely the result of viral replication reactivation in the replicating cancer calls, causing an increased viral load and therefore a higher prevalence in cancer patients, compared to healthy subjects (Cotmore et al., 2014; Kishore and Kishor, 2014; Phan et al., 2016; Qiu et al., 2017). In addition, some parvoviruses are known to be oncolytic, a fact that strongly argues against our hypothesis that HBoV is involved in tumorigenesis (Marchini et al., 2015). In contrast, as an argument for our hypothesis, it has to noted that the group of Jianming Qiu from Kansas has shown that HBoV appears to be the first known autonomous parvovirus whose replication appears to be independent of the cell cycle, which is different from the parvoviruses mentioned above (Deng et al., 2016, 2017).

However, we could not detect HBoV in breast, cervical or renal tumors (Schildgen et al., 2013), although virus spreading in many cases occurs via viremia. Due to this fact the previous assumption becomes more unlikely. On the other hand it is also possible that HBoV could have an active or passive role in development of these tumors due to chronic inflammation, but because of lacking antibodies up to now it is not known which kind of viral proteins in the respective tumors are expressed and how they interact with cellular proteins. For this reason this hypothesis remains a matter of speculation but is supported by the increased prevalence of HBoV detection in higher tumor stages, the profibrotic and pro-cancerogenic cytokine patterns of HBoV infected in CuFi-8 air-liquid interphase cell cultures, HBoV positive BALs (Khalfaoui et al., 2016), and the recent results of a whole transcriptome sequencing approach of HBoV-infected HAE-cultures, which revealed beside others evidence for bocaviral involvement in tumorigenesis of head-and-neck cancers (Schildgen et al., submitted).

## Conclusion

With the restriction that a single study can only contribute to isolated aspects of carcinogenesis without providing the necessary comprehensive view, these observations lead to the conclusion that human bocavirus appears to be a factor with an unknown role in tonsil squamous cell cancer development. Although it remains to be investigated which cell type within the tonsil acts as a target of HBoV and its replication, it seems possible that the keratinocytes are able to propagate HBoV1. Independently, it turned out that HBoV and HPV occur in the same cells and their possible synergistic interaction with other environmental or inherited factors that eventually lead to malignant progression, will make it difficult if not impossible to classify solely a single agent as the causative factor (zur Hausen and de Villiers, 2014). Therefore, the present study is a good indicator for the direction of further studies, highly desirable to elucidate the role of HBoV in pathogenesis of oropharyngeal cancer. Finally, the fact that no increased MSI is observed in tonsillar tumors makes it unlikely that the virus interacts directly with the host genome but more likely persists in episomal form.

## Availability of Data and Materials

The datasets used and/or analyzed during the current study are available from the corresponding author on reasonable request.

## Author Contributions

MH and VS performed culturing and infection experiments. MH and OS performed and analyzed FISH experiments. MH and SM were responsible for all clinical procedures. MB was responsible for all pathology-related diagnostics including tumor staging. OS and VS have planned the study. MH, OS, and VS wrote the manuscript. VS supervised the study. IF performed MSI testing and analyses.

## Conflict of Interest Statement

The authors declare that the research was conducted in the absence of any commercial or financial relationships that could be construed as a potential conflict of interest.

## Abbreviations

FFPE tissue: formaldehyde-fixed paraffin-embedded tissue
FISH: fluorescence *in situ* hybridization
Geq: genome equivalents
HBoV 1: human bocavirus 1
HBV: hepatitis B virus
HEK cells: human embryonic kidney cells
HPV: human papilloma virus
MMP-9: matrix-metalloproteinase 9
MOI: multiplicity of infection
MSI: microsatellite instability
